# G Protein Coupled Receptors in Embryonic Stem Cells: A Role for Gs-Alpha Signaling

**DOI:** 10.1371/journal.pone.0009105

**Published:** 2010-02-08

**Authors:** Brian T. Layden, Marsha Newman, Fei Chen, Amanda Fisher, William L. Lowe

**Affiliations:** Department of Medicine, Division of Endocrinology, Metabolism and Molecular Medicine, Northwestern University Feinberg School of Medicine, Chicago, Illinois, United States of America; Ecole Normale Supérieure de Lyon, France

## Abstract

**Background:**

Identification of receptor mediated signaling pathways in embryonic stem (ES) cells is needed to facilitate strategies for cell replacement using ES cells. One large receptor family, largely uninvestigated in ES cells, is G protein coupled receptors (GPCRs). An important role for these receptors in embryonic development has been described, but little is known about GPCR expression in ES cells.

**Methodology/Principal Findings:**

We have examined the expression profile of 343 different GPCRs in mouse ES cells demonstrating for the first time that a large number of GPCRs are expressed in undifferentiated and differentiating ES cells, and in many cases at high levels. To begin to define a role for GPCR signaling in ES cells, the impact of activating Gs-alpha, one of the major alpha subunits that couples to GPCRs, was investigated. Gs-alpha activation resulted in larger embryoid bodies (EBs), due, in part, to increased cell proliferation and prevented the time-related decline in expression of transcription factors important for maintaining ES cell pluripotency.

**Significance/Conclusions:**

These studies suggest that Gs-alpha signaling contributes to ES cell proliferation and pluripotency and provide a framework for further investigation of GPCRs in ES cells.

## Introduction

In embryonic stem (ES) cells, receptor mediated signaling pathways are important for maintaining pluripotency [1], [2], a state characterized by the ability of ES cells to differentiate into cell types from all three germ layers [2], [3]. Signaling through the leukemia inhibitory factor (LIF) receptor in murine ES cells and a bone morphogenetic factor (BMP) receptor in human ES cells is important for pluripotency [2], [4], although additional pathways play critical roles [5]. Similarly, ES cell differentiation pathways are typically directed, in part, by sequential activation of receptor-mediated intracellular signaling pathways by peptides and other molecules [6], [7], [8]. Improved understanding of receptor-stimulated signaling pathways that contribute to ES cell pluripotency and differentiation will ultimately facilitate regenerative therapies.

G protein coupled receptors (GPCRs) are a large class of transmembrane receptors that transmit extracellular signals into cells by coupling to guanine nucleotide binding proteins (G proteins) and play a key role in many complex biological processes, including development [9]–[11]. For example, roles for apelin and its corresponding GPCR in cardiovascular development, GPR161 in lens development and left-right patterning, and PAR1, a protease-activated GPCR for thrombin, in endothelial signaling during development have been demonstrated [12]–[15]. Moreover, global knockout of some GPCRs and/or components of their signaling pathways are embryonic lethal or associated with significant developmental anomalies in mice and humans [16]. Despite the importance of GPCRs in development, with the exception of the Frizzled receptors [17], the role of GPCRs in ES cell pluripotency and differentiation has received little attention [18]. Since GPCRs are readily targetable sites for small molecules, as evidenced by their role as drug targets in humans [9], characterization of GPCRs and related signaling molecules in ES cells may facilitate developing new approaches to ES cell differentiation. Given that, one of the goals of this study was to examine GPCR expression in ES cells.

GPCRs signal through ∼20 internal G protein alpha subunits. One of these alpha subunits, the stimulatory alpha subunit of G-proteins (Gs-alpha), activates the cAMP pathway, a well described pathway which is important in multiple complex processes, including cell proliferation and differentiation. Gs-alpha is also important during development, as mice with heterozygous, homozygous, and tissue specific knockouts of Gs-alpha exhibit significant developmental abnormalities and lethality [19]. The role of Gs-alpha in stem cell biology was demonstrated more directly via its importance for haematopoietic stem cell engraftment in bone marrow [20]. Again, despite its importance in development, the role of signaling through Gs-alpha in ES cell biology has not been examined.

In this study, we profiled the expression of hundreds of GPCRs in both undifferentiated and differentiated ES cells using a GPCR-specific real time RT-PCR microarray specific for 343 GPCRs and data mining of RNA expression libraries. These studies demonstrated for the first time expression of novel GPCRs in undifferentiated and differentiated ES cells and, in some cases, differential expression during ES cell differentiation. We also tested whether signaling through Gs-alpha plays a role in ES cell biology finding that Gs-alpha activation leads to large embryoid bodies (EBs), in part by enhancing the proliferation rate of cells within EBs. We also tested whether signaling through Gs-alpha impacts ES cell pluripotency and differentiation, and demonstrated that this G protein signaling pathway alters the expression of transcription factors important for maintaining ES cell pluripotency.

## Methods

### ES Cell Culture

The R1 mouse ES cell line was used, and mouse embryonic fibroblasts (MEFs) were prepared as described previously [21]. Undifferentiated ES cells were grown on irradiated mouse MEFs with knockout-DMEM (Invitrogen, Carlsbad, CA) supplemented with 20% serum knockout serum replacement (Invitrogen), 10^3^ U/ml leukemia inhibitory factor (LIF), 0.1 mM non-essential amino acids, 2 mM glutamine, 500 U/ml penicillin/streptomycin, and 0.55 mM 2-mercaptoethanol [22]. ES cells were detached from the dishes using 0.05% trypsin-EDTA and then grown in suspension culture in 60×20 mm petri dishes (Nunc, Rochester, NY) at an initial density of ∼4×10^5^ cells/ml of knockout-DMEM without LIF to facilitate formation of EBs. Four days after initiating growth in suspension, medium changes were performed every 2 days. For experiments with cholera toxin (CTX), the medium was supplemented with CTX (1 µg/ml). EB formation was allowed to occur over a 4-day period, and, following EB formation, the medium was changed every 2 days. To generate feeder layer independent R1 ES cells, ES cells were grown on gelatin plates for 5–6 passages without MEFs in the presence of LIF as previously described [23]. For EB formation in hanging drops, ES cells were trypsinized and counted using standard protocols, and cells were either resuspended in medium without or with CTX (1 µg/ml). Drops (500 cells/20 µl drop) were placed and allowed to grow for 2 days. The EBs were then transferred individually into a 96 well plate (non-coated) for continued growth [24].

### Quantification of Embryoid Body Size

EBs were examined every 4 days using light microscopy, and images were obtained to determine EB diameter. Digital images were acquired using a Spot camera, and the accompanying image analysis software (Diagnostic Instruments, Inc., Sterling Heights, MI) attached to a Nikon Eclipse 50i microscope (Nikon, Tokyo, Japan) was used to process the images.

### Real Time RT-PCR

Cells were isolated and lysed using Trizol reagent (Invitrogen). Total RNA was isolated, DNA removed by DNAase I (Ambion, Applied Biosystems, Carlsbad, California) digestion, and cDNA was prepared with the iScript cDNA synthesis kit (BioRad, Hercules, CA). Samples were run at a 1/5 to 1/40 dilution using SYBR green master mix (Qiagen, Valencia, CA). Real time RT-PCR was performed on a 96 well iCycler iQ5 (BioRad). mRNAs encoding markers of pluripotency (Nanog, Oct4), ectoderm (E-Cadherin, Nestin), mesoderm (Brachyury, Mixl1), and endoderm (Sox17, Fox2A and Cxcr4) were examined by real time RT-PCR, and the level of the different mRNAs was normalized to the level of the housekeeping gene, beta-actin (see Table 1 for primer sequences). A comparative Ct (cycle time) analysis was utilized to determine fold change in RNA expression based on the ΔΔCt approach [25].

**Table 1:** Real-time RT-PCR primers (forward and reverse sequences).

Markers	Forward	Reverse
Nanog	ctcttcaaggcagccctgat	ccattgctagtcttcaaccac
Oct4	ggcgttctctttggaaaggtg	ctcgaaccacatccttctct
E-Cadherin	aaacttggggacagcaacatcag	tcttttggtttgcagagacaggg
Brachyury	catgtactctttcttgctgg	ggtctcgggaaagcagtggc
Mixl1	gcacgtcgttcagctcggagcagc	agtcatcctgggatccggaacgtgg
Sox17	ttggaacctccagtaagccag	agatgtctggaggtgctgctcatt
Fox2A	caggtcggggtcttgggagtgc	ggaggagggggccgaagaacc
Cxcr4	cgggatgaaaacgtccattt	atgaccaggatcaccaatcca
Beta-actin	accaactgggacgatatggagaaga	tacgaccagaggcatacagggacaa
Mc4R	acagcgagtctcagggaaaa	ttgaccagtctgctgtttgc
SSTR1	ctactgtctgactgtgct	atgggcaagataaccagtaat
LPA2	cactcagcctagtcaagacggtt	gcatctcgggaatataccact
GPR108	cgagctgacatccaactgaa	gatgaggaacaggaccagga
GLP-1	gggtctctggctacataaggacaac	aaggatggctgaagcgatgac

### GPCR RNA Arrays

A commercially-available TaqMan real time RT-PCR GPCR 384-well microarray (Applied Biosystems, Carlsbad, California) which allows the expression of mRNAs encoding GPCRs from 50 different subfamilies (343 receptors, not including the odorant, olfactory, gustatory and pheromone receptors) to be quantified was used with RNA extracted from day 4 and 20 EBs, as well as undifferentiated ES cells grown for 5 passages in a feeder independent culture. For this assay, cDNA was prepared as described above, and each port was loaded with cDNA (from 1 µg of RNA) and the TaqMan Gene Expression Master Mix (Applied Biosystems) according to the manufacturer's instructions. The plate was analyzed on the 7900HT ABI PRISM in the Northwestern University Genomics Core. The results of the assay were analyzed using SDS2.3 and RQ Manager 1.2 software provided by Applied Biosystems. Cycle times were normalized to the house keeping gene beta-2 microglobulin. A comparative Ct approach was utilized to quantify the relative levels of mRNA [25] and computed using the RQ 1.2 software. Relative expression level was calculated as 2 ^(Ct GPCR–Ct control)^×10^5^. The results of each individual GPCR were examined, and if the sample had a calculated threshold less than 0.1, it was determined to be undetectable. In these instances, for data processing purposes, the cycle number was set at 40.0.

### Western Blot Analysis

EBs were isolated in RIPA cell lysis buffer containing a cocktail of protease inhibitors (Protease Inhibitors and Phosphatase Inhibitors mixes used as recommended, Calbiochem, LaJolla, CA) at different time points. Primary antibodies were obtained from Cell Signaling, except for antibodies directed against Nanog (Applied Biosystems), melanocortin-4 receptor (MC4R, Santa Cruz, Santa Cruz, CA) and Gs-alpha (Calbiochem). The protein content of the lysate was determined using the Coomassie blue protein assay. Thirty micrograms of protein were separated by 10% SDS-PAGE and transferred to a polyvinylidene difluoride membrane in a semidry apparatus. The membranes were blocked in 20 mM Tris (pH 7.6), 137 mM NaCl, and 0.1% Tween 20 (TBS-T) and 4% nonfat dry milk for 60 min at room temperature. Membranes were incubated overnight at 4°C in TBS-T containing 5% BSA and primary antibody, washed three times for 10 min at room temperature in TBS-T, and incubated for 60 min at room temperature in TBS-T containing 4% nonfat dry milk and secondary antibody (1∶4000 dilution). After three washes in TBS-T, immunoreactive bands were detected using the enhanced chemiluminescence detection system from Amersham Pharmacia Biotech (Arlington Heights, IL), according to the manufacturer's instructions.

### Immunofluorescence Analyses

EBs were isolated on different days over a 20 day period and fixed by washing with 1X PBS at room temperature (RT), incubating in 4% paraformaldehyde (PFA) for 30 min at RT, washing again with 1X PBS and then adding 80% ethanol prior to embedding. To quantify cells which expressed Nanog, EBs were sectioned every 5 µm. Three randomly-selected slides from different sample preparations were stained for both Nanog and DAPI, and the number of Nanog^+^ cells relative to DAPI^+^ cells was determined. Images covering the slide were obtained and systematically counted to determine the ratio of Nanog^+^/DAPI^+^ cells by an individual blinded to the experimental conditions and expected outcomes. The data are presented as an average of the percentage of Nanog^+^ cells relative to DAPI^+^ cells for each slide (average of three separate slides counted for each condition). A similar approach was used to determine the number of Ki67^+^ cells in EBs treated or not with CTX. Slides from three randomly-selected sections from different sample preparations were stained for both Ki67 and DAPI and the number of Ki67^+^ cells relative to DAPI^+^ cells in each section was determined.

### WST-1 Assay

EBs were isolated at the indicated time points, washed once with PBS, and resuspended in PBS with WST-1 (Roche, Hannheim, Germany). The EBs were then incubated and analyzed according to the manufacturer's instructions. Metabolically active cells were quantified by following absorbance at A450 [26].

### Statistical Analyses

Values are reported as the mean ± SEM. *P* values were calculated by Student's t-test with a significance level at *P*<0.05 using SigmaStat 3.1 software (SYSTAT Software, Inc., Richmond, CA).

## Results

### GPCRs Expression in ES Cells

**Figure 1:**
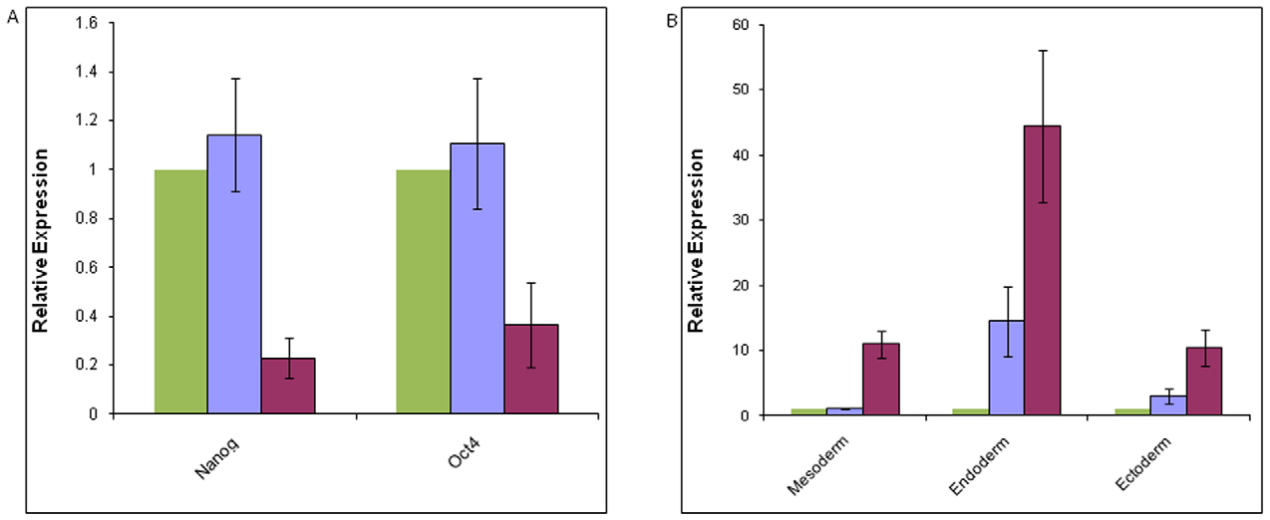
Changes in mRNA levels for markers of pluripotency and the three germ cell layers in undifferentiated ES cells relative to EBs at day 4 and day 20. (A) Relative changes in the level of mRNA encoding markers of pluripotency, Nanog and Oct4, during EB differentiation at day 4 (blue) and at day 20 (red) as compared to undifferentiated ES cells (green). Values are the mean ± SEM (n = 5 for Nanog and n = 4 for Oct4) of the relative level of mRNA in day 4 and day 20 EBs compared to the level in undifferentiated ES cells which was defined as 1.0. (B) Relative changes in the level of mRNA encoding markers of the three germ layers; mesoderm (Brachyury), endoderm (CXCR4), and ectoderm (Nestin) during EB differentiation, at day 4 (blue) and at day 20 (red), as compared to the level of mRNA in undifferentiated ES cells (green). Values are the mean ± SEM (n = 4 for Brachyury, n = 3 for CXCR4, and n = 3 for Nestin) of the level of mRNA in day 4 and day 20 EBs compared to the level in undifferentiated ES cells, which was defined as 1.0.

Initial studies examined the differential expression of GPCRs in ES cells from day 4 and 20 EBs. EBs at day 4 contain mostly undifferentiated ES cells as compared to day 20 EBs. This is evident by comparing the level of expression of mRNA encoding Nanog and Oct4 in day 4 EBs and undifferentiated ES cells. The levels were essentially the same, while by day 20, the level of mRNA encoding Nanog and Oct4 had decreased significantly compared to the level in day 4 EBs. In contrast, between day 4 and day 20, the expression of mRNAs characteristic of the three germ layers increased (Figure 1A–B).

**Figure 2:**
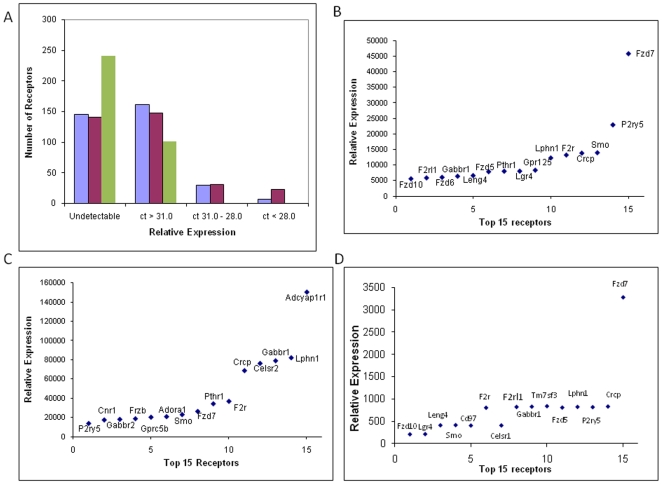
GPCR expression in ES cells using a real time RT-PCR microarray. (A) The level of mRNA encoding GPCRs from day 4 (blue) and day 20 (red) EBs and undifferentiated ES cells (green) was categorized as undetectable, low, medium, and high. The cycle number (Ct) for the low, medium, and high groups were greater than 31.0, 31.0–28.0, and less than 28.0, respectively. Ct values for each GPCR were the average of the results from 2 independent experiments. (B) Receptors with the highest level of expression in day 4 EBs are indicated with their corresponding relative level of expression and gene name. The relative expression is presented as 2 ^Ct (GPCR)–Ct (housekeeping gene)^×10^5^. (C) Receptors with the highest level of expression in day 20 EBs are indicated with their corresponding relative level of expression and gene name. The relative expression is presented as 2 ^Ct (GPCR)–Ct (housekeeping gene)^×10^5^. (D) Receptors with the highest level of expression in undifferentiated ES cells are indicated with their corresponding relative level of expression and gene name. The relative expression is presented as 2 ^Ct (GPCR)–Ct (housekeeping gene)^×10^5^.

To profile the expression of mRNAs encoding 343 non-odorant GPCRs in ES cells at day 4 and 20, a real time RT-PCR GPCR-specific microarray was used (see Text S1 for the list of GPCR genes in the array and Text S2 for the raw expression data). To categorize the general expression level of the different genes, RNAs were assigned to one of the following groups: undetectable (see Methods for description), low (cycle number >31.0), moderate (cycle number between 31.0 to 28.0), and high (cycle number less than 28.0) expression. The ranges for these categories were defined based on the knowledge that frizzled receptors (Fzd 1–10) are expressed in mouse as well as in human ES cells [27]. The mean cycle number of Fzd receptors 1–10 in our microarray data from day 4 EBs were 30.8, 28.6, 29.2, 31.4, 28.0, 28.4, 25.5, 28.7, 34.7, and 28.5 (n = 2), respectively. We arbitrarily defined our categories to have a majority of these Frizzled receptors in the moderate to high expression level. As seen in Figure 2A, 161 and 148 receptors exhibited low expression at day 4 and day 20, respectively, and 30 and 31 receptors exhibited moderate expression at day 4 and day 20, respectively. At day 4, 7 receptors were in the high expression category. At day 20, 23 were in the high expression category. 145 and 141 receptors were undetectable at day 4 and day 20, respectively.

The 15 receptors with the highest level of expression in EBs on days 4 and 20 are shown in Fig. 2B and 2C. On day 4, five frizzled receptors (Fzd7, Smo, Fzd5, Fzd6, and Fzd10) and, on day 20, three frizzled receptors (Smo, Fzd7, Fzdb) were highly expressed. The function of the other highly expressed receptors in day 4 and 20 EBs is not clear. Among the 15 most highly expressed receptors, seven (Lpn1, Gabbr1r, Crcp, Fzd7, F2r, Pthr1, Smo, P2ry5) receptors were highly expressed at both day 4 and day 20. Although some receptors were highly expressed at both time points, many receptors demonstrated a dramatic difference in expression between day 4 and day 20. Receptors which were up- or down-regulated between day 4 and 20 are listed in Tables 2 and 3, respectively.

**Table 2:** GPCRs that were upregulated in day 20 compared to day 4 EBs.

Family	Gene	Fold Change	Family	Gene	Fold Change
PACAP	Adcyap1r1	306	Orphans (cont)	Gpr87	7
Adenosine	Adora1	56		Gpr26	12
	Adora2a	10		Lphn1	7
Angiotensin	Agtr2	18		Lphn3	73
Adrenoceptor	Adra2a	33	Glutamate	Grm2	6
	Adrb1	18		Grm3	202
	Avpr1b	11		Grm4	41
Vasopressin	CCr10	12		Grm5	135
	Crcp	5		Grm8	169
Calcitonin	Celsr2	79	Orexin	Hcrt1	4
Cadherin	Celsr3	6	Histamine	Hrh1	9
Acetylcholine	Chrm3	9		Hrh2	6
	Chrm5	68		Hrh3	13
Cannaboid	Cnr1	13	Serotonin	Htr1b	4
Dopamine	Drd2	20		Htr2a	11
Endothelin	Ednrb	34		Htr2c	6
Frizzled	Frzb	83		Htr4	35
	Fzd1	12	Kisspeptins	Kiss1r	8
GABA	Gabbr1	12	Leucine-rich	Lgr5	36
	Gabbr2	1083		Lgr6	4
Glucagon	Ghsr	9	Melanocortin	Mc4r	377
	Gipr	6		Mchr1	92
Orphans	Gpr123	5	Neuropeptide FF	Npffr1	10
	Gpr34	5	Opoiod	Oprk1	6
	Gpcr5b	5		Oprl1	6
	Gpr139	236		Oprm1	10
	Gpr22	131	Purinergic	P2ry1	6
	Gpr45	453	PTH	Pthr1	4
	Gpr50	23	Rhodopsin	Rrh	12
	Gpr149	5	Somatostatin	Sstr1	422
	Gpr156	32		Sstr2	5
	Gpr162	29		Sstr5	5
	Gpr176	9	Tachykinin	Tacr1	86
	Gpr160	4		Tacr3	82
	Gpr62	8	Luteinizing	Lhcgr	26
	Gpr63	6	Neuromedin U	Nmur2	10
	Gpr85	6			

GPCRs that showed a greater than 4 fold increase in RNA level are listed. The genes are categorized into their family names (based roughly on IUPHAR categories), and their fold change is indicated. The data represent the average of the results from two independent experiments.

**Table 3:** GPCRs that were down-regulated in day 20 compared to day 4 EBs.

Family	Gene	Fold Change
Chemokine	Ccr2	0.04
	Cxcr4	0.04
	Ccr7	0.14
Lysophospholipid	Edg4	0.01
	Edg5	0.01
Thrombin	F2rl1	0.01
Frizzled	Fzd5	0.01
Endothelin	Ednra	0.01
Glucagon	Glp1r	0.25
	Gcgr	0.04
Orphans	Gpr103	0.01
	Gpr41	0.09
	Gpr108	0.01
	Gpr1	0.07
	Gpr124	0.25
	Gpr135	0.22
	Gpr133	0.01
	Gpr146	0.04
	CD97	0.01
Leukotriene	Mass1	0.07
Somatostatin	Sstr3	0.07
Prostanoid	Tbxa2r	0.21
Free fatty acid	Ffar1	0.12

GPCRs that showed a greater than 4 fold decrease in RNA level are listed. The genes are categorized into their family (based roughly on IUPHAR categories), and their fold change is indicated. The data represent the average of the results from two independent experiments.

**Figure 3:**
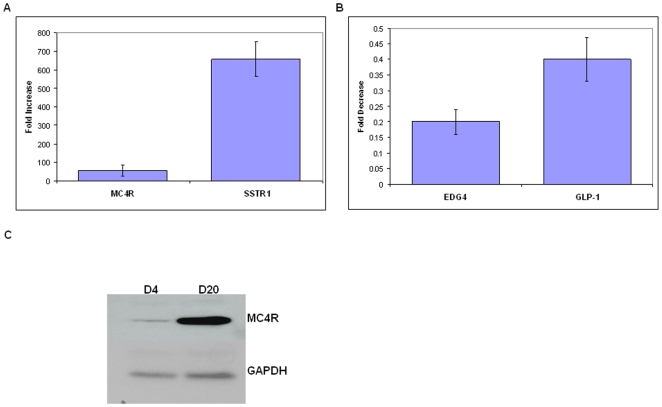
Verification of GPCR mRNA levels using real time RT-PCR. (A–B) Relative changes in the level of mRNA encoding MC4R and SSTR1(A) and the EDG4 and GLP-1 receptors (B) in day 20 compared to day 4 EBs, as determined by real time RT-PCR (mean ± SEM, n = 3 or 4). (C) Western blot analysis of MC4R protein expression (representative of the results from three independent experiments) in extracts of day 4 (D4) and day 20 (D20) EBs. Following hybridization with antibodies directed against the above receptors, the blots were stripped and re-probed with antibody against GAPDH.

Real time RT-PCR was used to verify the change in expression level of some of these receptors. As seen in Fig. 3, MC4R and SSTR1 mRNA levels were up regulated from day 4 to 20 (Fig. 3A), while LPA2, GPR108, and GLP-1 receptor mRNA levels were down regulated (Fig. 3B). These changes are consistent with those observed in the microarray experiments. To determine if these changes in mRNA levels were reflected by a change in the level of protein, Western blot analyses were performed to examine the protein levels for one of these receptors, MC4R. Consistent with the change in mRNA level, the expression of MC4R was increased in day 20 compared to day 4 EBs (Fig. 3C).

Because some cells in day 4 EBs have likely undergone some degree of differentiation, as reflected by the increased level of an mRNA encoding a protein characteristic of endoderm in day 4 EBs compared to undifferentiated ES cells (Figure 1B), we performed a similar analysis on undifferentiated ES cells grown in a feeder free system. Overall, the relative expression of the GPCRs was much lower in undifferentiated ES cells than in day 4 and 20 EBs. Using the classification scheme in Figure 2A for the undifferentiated ES cells, no GPCRs were in the high expression group, 1 GPCR was in the moderate expression group, and 100 were in the low expression group. The remaining GPCRs were not detectable. No GPCRs exhibited increased expression (greater than 4 fold) in undifferentiated ES cells compared to day 4 EBs. The 15 GPCRs exhibiting the largest increase in day 4 EBs compared to undifferentiated ES cells are shown in Table 4.

**Table 4:** The 15 GPCRs that exhibited the largest change in expression in day 4 EBs compared to undifferentiated ES cells.

Gene	Fold Change
Gpr133	1417
Ccr2	683
Cxcr4	278
Gpr26	247
Fzd6	242
Adra2b	210
Fzd2	149
Gpr23	138
Mass1	137
Oprd1	122
Adora1	121
Sstr3	117
Vipr2	85
Adra2a	76
Ppyr1	75

The data represent the average of the results from two independent experiments.

When comparing the 15 GPCRs that were the mostly highly expressed in undifferentiated ES cells and day 4 EBs, 12 of the 15 receptors were the same (Figure 2D). Three receptors; TM7sf3, Cesr1, and CD97l, were among the 15 most highly expressed GPCRs in undifferentiated ES cells but not day 4 EBs. However, each of these receptors were expressed at relatively high levels in day 4 EBs (among the top 40 receptors overall). Similarly, the GPCRs that were among the 15 most highly expressed receptors in day 4 EBs and not in this group in undifferentiated ES cells (Gpr125, PTH1r and Fzd6) were all expressed in undifferentiated ES cells. In general, GPCR expression in undifferentiated ES cells and day 4 EBs was similar (see Text S2 for the undifferentiated ES cell raw expression data).

Searching expressed sequence tag (EST) databases is an alternative *in silico* approach for examining expression profiles of GPCRs. To further support the results of our microarray analyses, we examined the expression of GPCRs in a database generated by Cloonan et al., which included libraries from undifferentiated mouse ES cells and EBs at day 4 of formation. Examination of the Gene Expression Omnibus (GEO) database (GSE10518) for the 343 GPCRs assayed in our real time RT-PCR microarray demonstrated that 30 were present in the GEO database (Table 5). All of these receptors were also detected using the GPCR microarray in day 4 EBs. Most receptors that were detected by this data mining approach were in the moderate and high expression categories as determined in the real time RT-PCR microarray. Interestingly, these same 30 receptors were also present in a human ES cell EST database [28]. These data also demonstrate good overlap with our GPCR expression data in undifferentiated ES cells. Of the 17 GPCRs in the GEO database which were reported to be expressed only in undifferentiated ES cells, 15 were detected by the GPCR microarray using the undifferentiated ES cells (missing were Gpr19 and Gpr23).

**Table 5:** Data mining of a whole genome RNA expression library (GSE10518) from ES cells at day 0 and day 4 of EB formation for GPCRs*.

Day 0	Day 0 & Day 4	Day 4
Cd97 (l,h,u)	Celsr3 (l,h,h)	Adcyap1r1 (l,m,h)
Tm7sf3 (l,h,h)	Crcp (l,h,h)	Adra2a (u,m,h)
Fzd5 (l,h,l)	Edg2 (l,h,h)	Adra2b (u,m,m)
Gabbr1 (l,h,h)	Edg4 (l,h,l)	Ednra (l,l,u)
	Edg5 (l,h,u)	Frzb (l,m,h)
	F2r (l,h,h)	Lgr5 (u,l,m)
	F2rl1 (l,l,h)	Prokr1 (l,l,l)
	Fzd7 (m,h,h)	Pthr1 (l,h,h)
	Gpr108 (l,l,h)	Agtrl1 (u,l,m)
	Gpr125 (l,h,h)	Celsr1 (l,h,h)
	Gpr137 (l,h,h)	Cxcr4 (u,m,l)
	Gpr19 (u,l,u)	Fzd3 (l,h,h)
	Gpr23 (u,m,m)	Gpr124 (l,h,m)

* Day 0 represents undifferentiated ES cells prior to EB formation and Day 4 represents EBs analyzed at day 4 of EB formation. This database was mined for the GPCRs included in the real time RT-PCR array (see Text S2). Receptors were identified as expressed at day 0 alone, day 0 and day 4 both or day 4 alone. In parenthesis, next to each gene name is the corresponding expression level determined by the GPCR specific real time RT-PCR microarray for undifferentiated ES cells and day 4 and day 20 EBs (h =  high, m =  medium, l =  low, and u =  undetectable).

### Gs-Alpha Signaling in Mouse ES Cells

**Figure 4:**
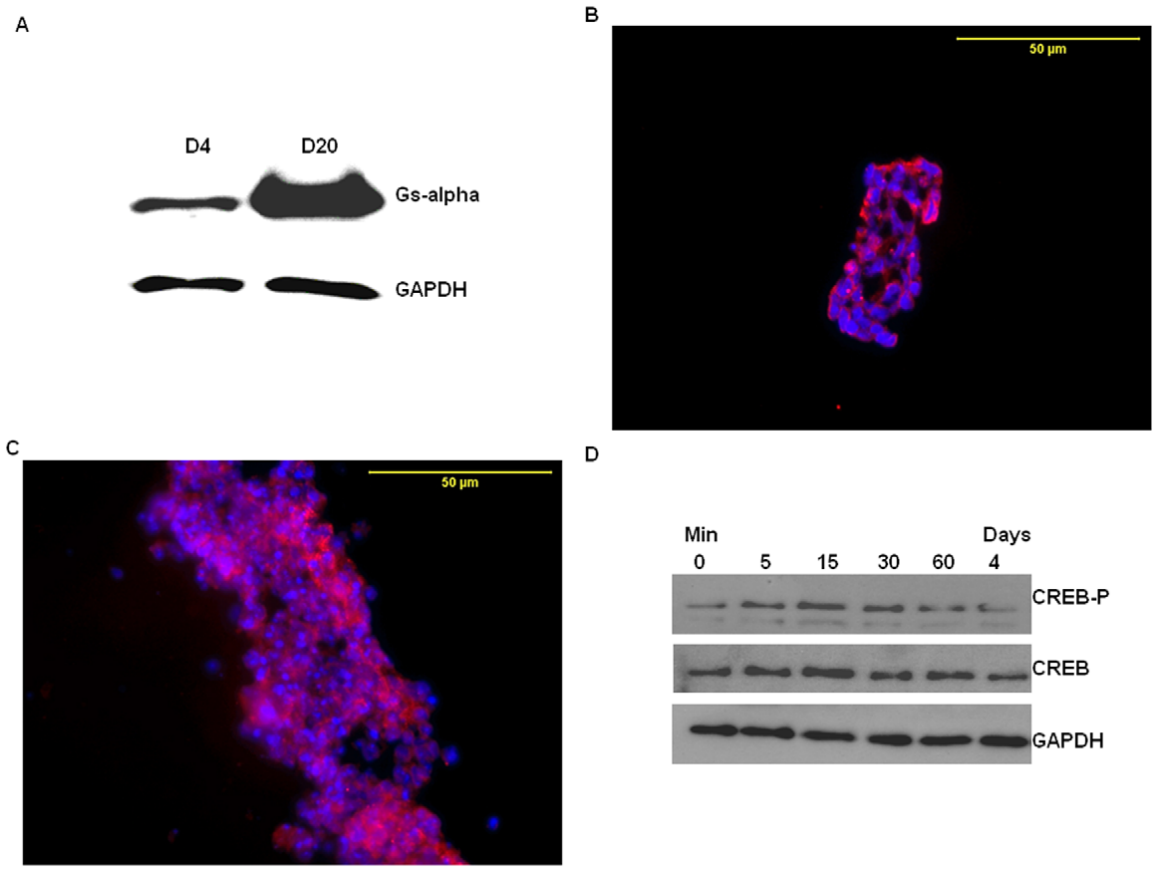
Gs-alpha is expressed and functional in mouse ES cells forming EBs. (A) Representative immunoblot of Gs-alpha expression in extracts of day 4 (D4) and day 20 (D20) EBs. The findings are representative of the results of three independent experiments. After hybridization, the blots were stripped and re-probed with antibody against GAPDH. (B–C) Immunohistochemical localization of Gs-alpha in EBs. Day 4 (B) and day 20 (C) EBs were immunostained with an antibody to Gs-alpha (red), and nuclei were stained with DAPI (blue), magnification was 40×. (D) Immunoblot of phospo-CREB in day 4 EBs treated with 1 µg/ml CTX for 0, 5, 15, 30 and 60 mins, as well as 4 days. Immunoblots for total CREB and GAPDH using the same samples are also shown. The results are representative of the results from 3 independent experiments.

**Figure 5:**
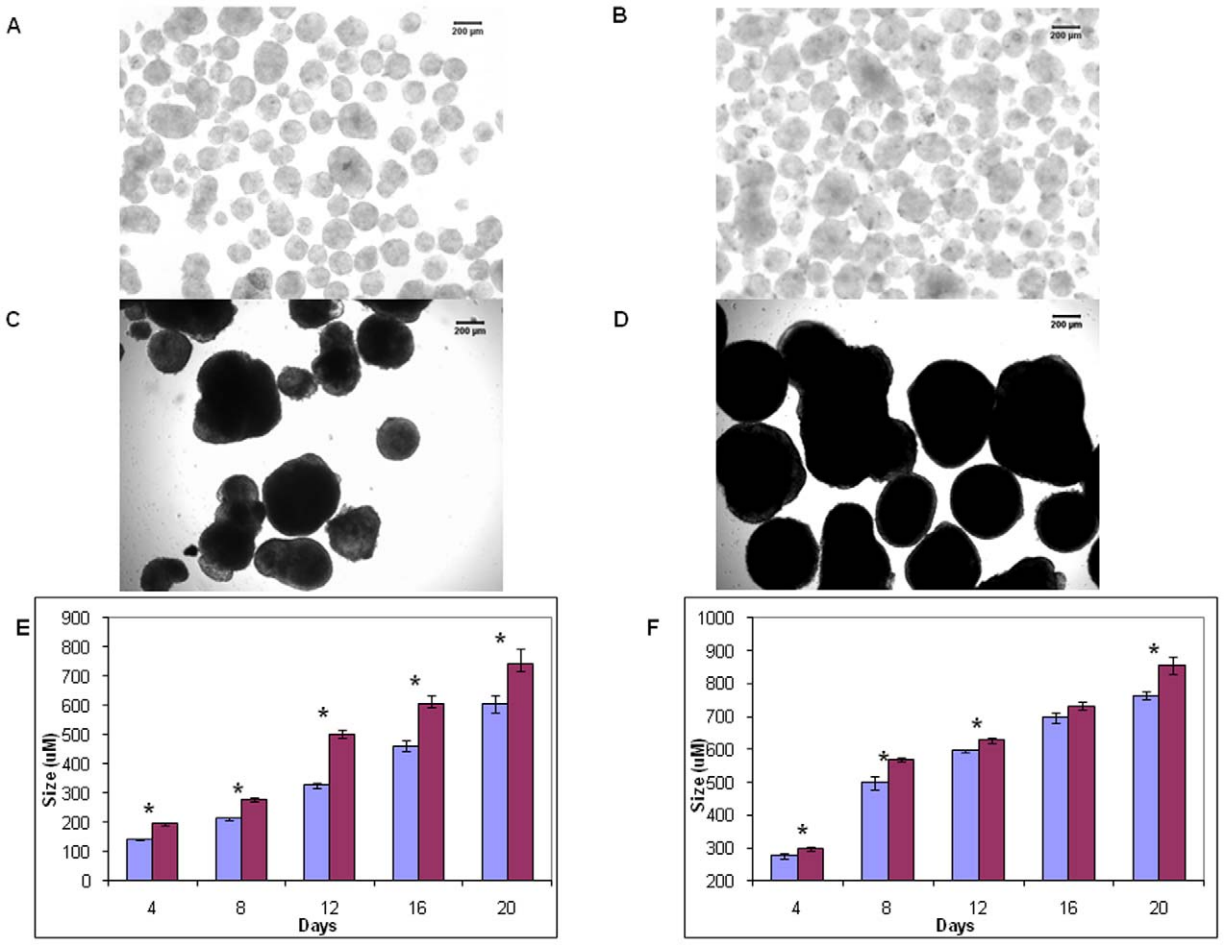
The effect of Gs-alpha activation by CTX on EB size. (A–D) EB formation in the absence (A,C) or presence (B,D) of 1 µg/ml CTX at day 4 (A,B) and day 12 (C,D). (E) A comparison of the diameter (mean ± SEM) of EBs incubated in the absence (blue bars) and presence (red bars) of 1 µg/ml CTX over a 20 day period. One random image was taken of each sample of EBs, and the greatest horizontal diameter for each EB in the horizontal plane was measured. A total of 4 to 9 (independently prepared) EB preparations were studied at each time point. *, *P*<0.05 compared to control, untreated EBs. Total number of EBs counted for each condition was for day 4 (control, CTX; 417, 395), day 8 (346, 195), day 12 (255, 129), day 16 (58, 50) and day 20 (36, 25). (F) A comparison of the diameter (mean ± SEM) of EBs produced by the hanging drop method and incubated in the absence (n = 10 at each time point, blue bars) and presence (n = 11 at each time point, red bars) of 1 µg/ml CTX over a 20 day period. An image was taken on each individually grown EB, and the greatest horizontal diameter for each EB in the horizontal plane was measured. *, *P*<0.05 compared to control, untreated EBs.

Having established that multiple GPCRs are expressed in ES cells and some are differentially expressed during differentiation, we next sought to investigate the role of Gs-alpha signaling pathways in differentiating ES cells. Prior to investigating the effects of CTX on ES cells, Gs-alpha expression and function in the ES cells was confirmed. Western blot analyses demonstrated that Gs-alpha is expressed in differentiating ES cells during EB formation with expression evident in EBs at both day 4 and 20 (Fig. 4A). Further, immunohistochemical analyses demonstrated Gs-alpha expression in most cells within EBs at day 4 and 20 with no evidence for regional localization of expression within the EBs (Fig. 4B and 4C). To verify that the Gs-alpha pathway is functional in differentiating ES cells, EBs were treated with 1 µg/ml CTX, which is a toxin that ADP-ribosylates Gs-alpha, resulting in permanent activation of Gs-alpha, cAMP generation, and, ultimately, activation of the transcription factor, cAMP-response element binding protein (CREB). The response of the ES cells to CTX was tested by examining CREB phosphorylation in day 4 EBs. As seen in Fig. 4D, CTX treatment of day 4 EBs increased CREB phosphorylation, consistent with the presence of a functional Gs-alpha pathway. Next, we examined the impact of CTX treatment on EB morphology. We found that EBs treated with CTX were consistently larger than control, untreated EBs between days 4 and 20 (Fig. 5A-D). When the diameter of the EBs was determined, a significant increase in the diameter of CTX-treated compared to control EBs was observed (Fig. 5E). Because this finding could reflect CTX leading to increased EB aggregation and, thus, larger EBs, we explored this possibility by growing individual EBs using the hanging drop method [24]. As is apparent, when this method was used, a similar trend toward larger EBs was seen in the CTX–treated compared to control group (Fig. 5F).

**Figure 6:**
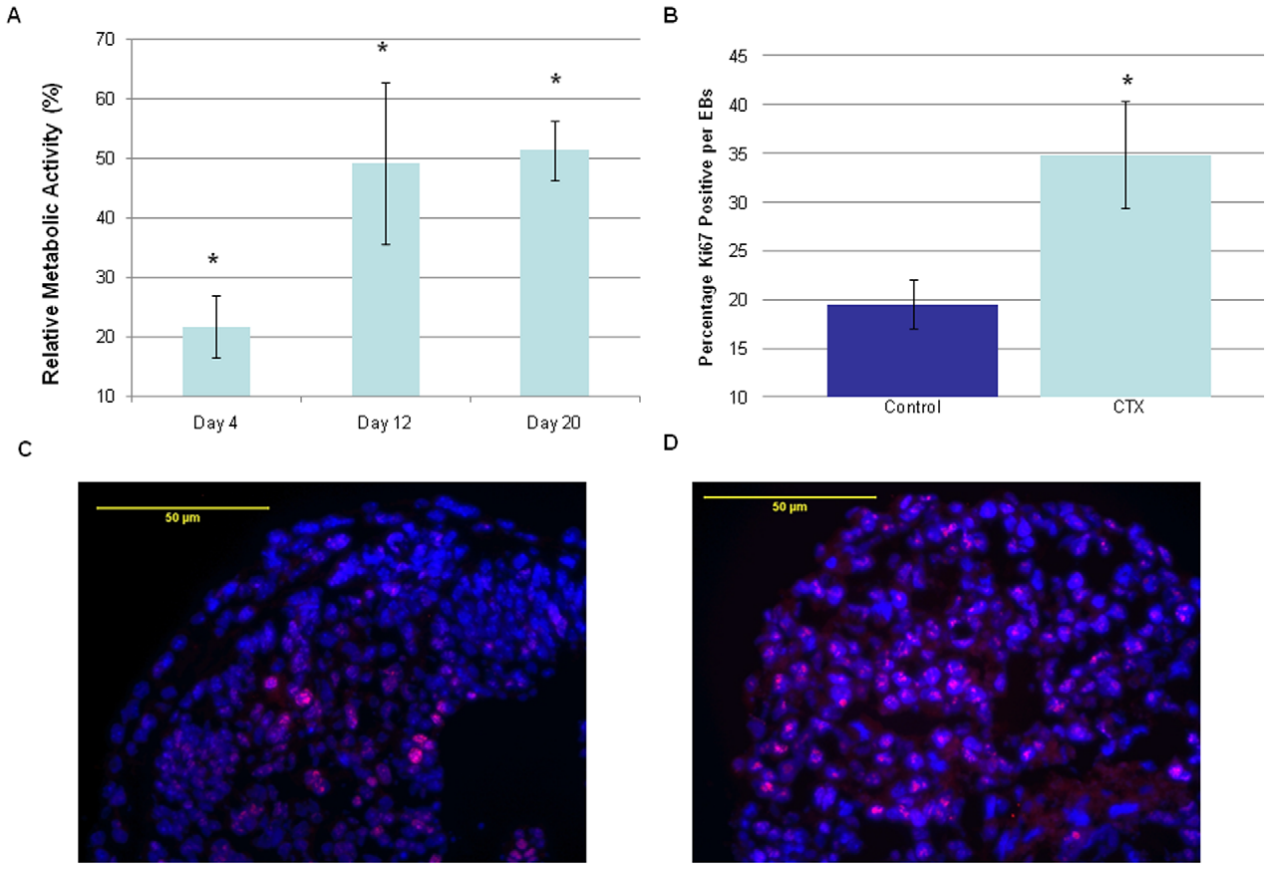
Effect of Gs-alpha activation on cell proliferation in EBs. (A) Cell metabolic activity in EBs over a 20 day period of CTX treatment was evaluated, and the percentage change in the CTX-treated compared to control cells was determined in three independent samples at each time point. The values represent the relative percentage change in absorbance A450 in the CTX-treated EBs as compared to the control EBs, and the percentage change is shown. *, *P*<0.05 compared to control, untreated EBs. (B) The percentage of Ki67^+^ cells in CTX-treated and control EBs at day 20 relative to DAPI^+^ positive cells was determined as described in the *Methods*. *, *P*<0.05 compared to control cells. Representative image of immunohistochemical localization of Ki67 (red) in control (C) and CTX-treated (D) day 20 EBs is shown. EBs were immunostained with an antibody to Ki67, and nuclei were stained with DAPI (blue).

Because the results of the above studies suggest that EBs grown in the presence of CTX are larger, we examined whether cell proliferation was increased in CTX-treated compared to control EBs. To do this, two complementary approaches were used. First, the WST-1 assay, an indicator of metabolic activity and, indirectly, proliferation, was used. As seen in Fig. 6A, CTX-treated EBs over 20 days exhibited increased metabolic activity compared to control EBs. Next, an immunohistochemical analysis was used to compare the expression of Ki67, a marker of cell proliferation, in CTX-treated and control day 20 EBs. As is apparent, the percentage of Ki67-positive cells in the EBs was higher in the CTX-treated compared to control EBs at day 20 (Figure 6B–D). Overall, these data suggest that CTX leads to increased cell proliferation in EBs.

**Figure 7:**
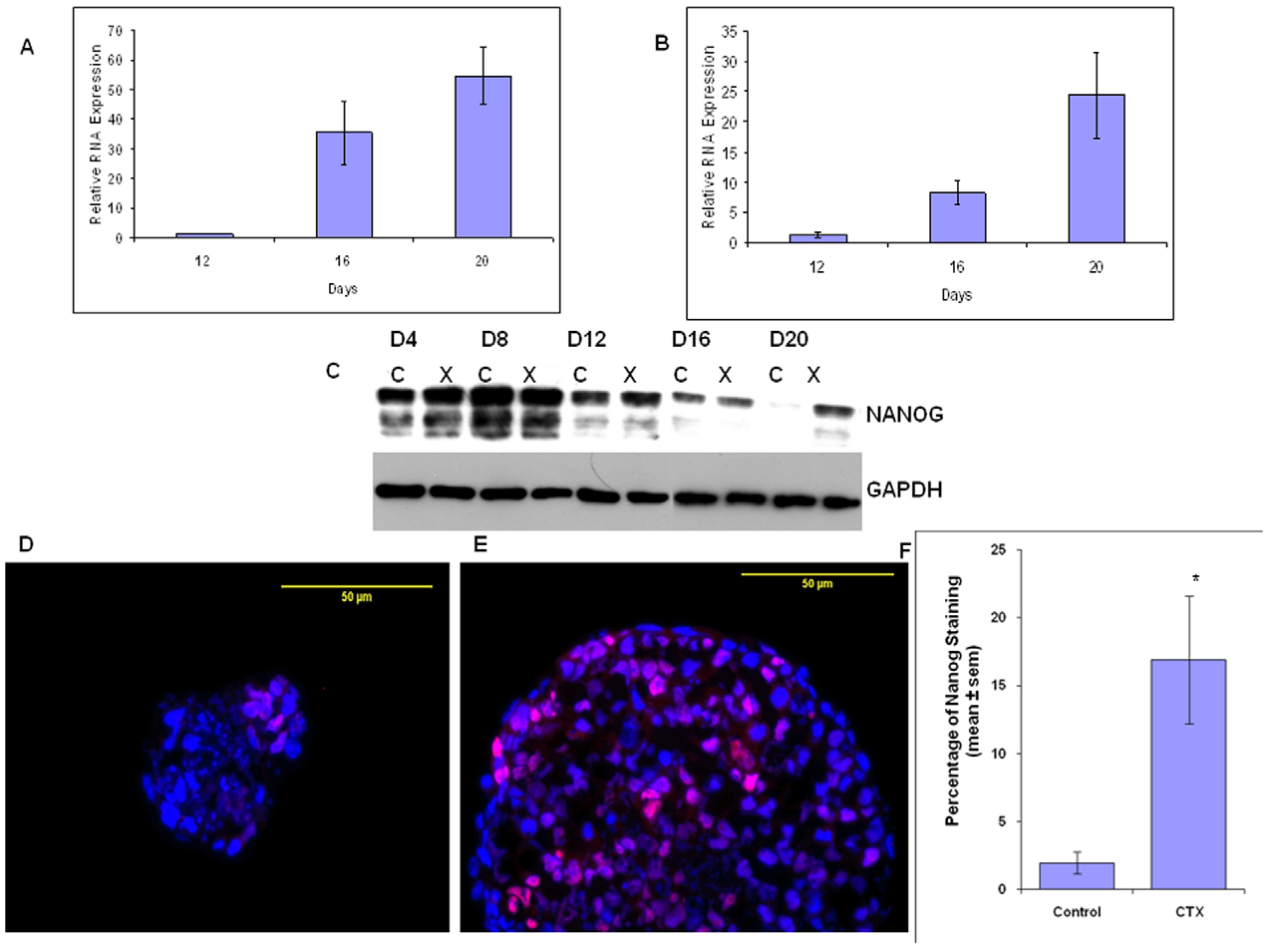
Effect of Gs-alpha activation on Nanog and Oct4 expression. (A–B) Fold changes in the level of Nanog (A) and Oct4 (B) mRNA in CTX-treated compared to control EBs at day 12, 16, and 20 as determined by real time RT-PCR. Values are the mean ± SEM (n = 3 for Nanog and n = 4 for Oct4). (C) Western blot analysis of Nanog expression in CTX-treated (X) compared to control cells (C) at different time points (D4, D8, D12, D16, D20). The blots were stripped and re-probed for GAPDH. The results are representative of the results of three independent experiments. (D–E) Immunohistochemical localization of Nanog (red) in control (D) and CTX-treated (E) day 20 EBs. EBs were immunostained with an antibody to Nanog (red), and nuclei were stained with DAPI (blue). The images are representative of the results of three independent experiments. (F) The percentage of Nanog^+^ cells in CTX-treated and control EBs at day 20 relative to DAPI^+^ positive cells was determined as described in the *Methods*. *, *P*<0.05 compared to control cells.

To determine whether Gs-alpha activation had an effect on the expression of proteins characteristic of pluripotent and differentiating ES cells in EBs, the effect of CTX treatment on the expression of markers of pluripotency and differentiation was examined. As described in the Methods, ES cells were grown in suspension culture for EB formation either in the presence or absence of CTX. For these studies, RNA was extracted from control and CTX-treated EBs at different time points between days 12 and 20, and the expression of mRNAs encoding proteins important for ES cell pluripotency or differentiation was quantified by real time RT-PCR. As can be seen in Fig. 7, the level of mRNA encoding transcription factors important for pluripotency, Nanog (Fig. 7A) and Oct4 (Fig. 7B), was increased in CTX-treated compared to control EBs at each time point.

As the change in the level of Nanog mRNA was more pronounced, subsequent studies focused on determining whether the change in Nanog mRNA levels was accompanied by a change in protein levels. Western blot analyses using protein lysates from control and CTX-treated EBs harvested at time points between day 4 and 20 demonstrated a decline in Nanog levels in control EBs while Nanog expression in CTX-treated EBs tended to be maintained (Fig. 7C). This trend of a greater decline in Nanog in the control EBs as compared to the CTX-treated EBs is also reflected in the RNA expression as seen in the cycle number (mean ± SD) at each of these time points (day 12 control vs CTX, 27.8±0.3 vs 28.2±0.2; day 16 control vs CTX, 32.8±1.0 vs 26.7±0.3; day 20 control vs CTX, 32.5±0.6 vs 27.6±0.7). Overall, Nanog mRNA and protein levels were relatively stable between day 12 and 20 in the CTX treated group, while declining in control EBs during this same time period. Immunohistochemical analyses demonstrated that the number of Nanog^+^ cells was increased in CTX-treated compared to control EBs in day 20 EBs (Fig. 7D–F). Taken together, the results of the above studies suggest that activation of Gs-alpha pathway helps to maintain expression of transcription factors important for pluripotency in ES cells.

The expression of markers of early differentiation was also examined by real time RT-PCR. The level of mRNAs encoding proteins present in cells differentiating along ectodermal (E-Cadherin), mesodermal (Brachyury, Mixl1), and endodermal (Sox17, Fox2a and CXCR4) pathways demonstrated no substantial difference in CTX-treated compared to control EBs between 12 and 20 days (Figure S1).

## Discussion

To explore the expression profile of GPCRs in ES cells, two main approaches were used, real time RT-PCR microarrays and data mining of EST libraries specific to ES cells. Interestingly, GPCRs represent only a small portion of the genes in ESTs relative to their representation in the whole genome [29]. Thus, these databases are thought to under represent the number of expressed GPCRs [27]. By examining an EST database created from mostly undifferentiated ES cells, 30 GPCRs were detected (Table 5). All of these receptors were also expressed in our real time RT-PCR microarrays at one of the time points evaluated in our study (undifferentiated ES cells or EBs at day 4 and day 20). The in silico data alone indicate that multiple GPCRs are expressed in ES cells. But, as noted, this probably under represents the GPCRs expressed in ES cells. Indeed, our real time RT-PCR microarray data demonstrate that a large number of GPCRs are expressed not only in undifferentiated ES cells but in differentiating ES cells in EBs as well. Moreover, a large number of these GPCRs are differentially expressed during ES cell differentiation in EBs. Interestingly, there is much lower overall expression of GPCRs in undifferentiated ES cells as compared to EBs at either day 4 or day 20. Despite these differences, the particular GPCRs that are expressed in undifferentiated ES cells are similar to those in day 4 EBs. Taken together, these findings demonstrate that a broad range of GPCRs are expressed in ES cells and likely play an important role in ES cell biology.

Based on previous embryological data, a number of the GPCRs were expected to be expressed at higher levels during the course of ES cell differentiation in EBs. For example, on day 4, two receptors from the proteinase-activated receptor family (PAR), F2r and F2rl1, were highly expressed. F2r, also known as PAR1, has been shown to have an important role in early embryonic development, as global knockout results in lethality at mid-gestation due to bleeding complications [15]. Another receptor that was highly expressed in our assays, GPR125, has been identified as a marker of germ-line progenitors [27], [30], although its role in ES cell differentiation is not clear. LGR4, another of the identified receptors, has a demonstrated role in development, as LGR4^−/−^ mice had decreased survival with most offspring dying by day 2 and exhibiting intrauterine growth retardation and abnormalities in kidney and liver development [31]. Even though the expression of these receptors is predicted in ES cells based on previous studies of development, the role of these GPCRs in mediating ES cell differentiation has not been specifically explored. The potential role of these and many GPCRs that were found to be expressed in ES cells warrants further study.

Activation of Gs-alpha by CTX consistently led to larger EBs over time. This effect is related, in part, to an increase in cell proliferation in CTX-treated compared to control EBs. One of the primary signaling pathways activated by Gs-alpha is the cAMP pathway. The role of cAMP in regulating proliferation in different cell lines has been previously studied. Interestingly, the effect of cAMP on proliferation is known to be cell type dependent [32], [33], [34]. In this study the overall effect of Gs-alpha activation and, presumably, the cAMP pathway was increased cell proliferation in the EBs. As cell differentiation along multiple different lineages occurs in the context of EBs, it is not clear at present whether the effect of CTX on cell proliferation was limited to undifferentiated or differentiating ES cells or was realized in both cell types. This will need to be addressed in future studies.

Our finding that Gs-alpha impacts the expression of transcription factors important for ES cell pluripotency is not unexpected considering the diverse roles of cAMP in multiple cell types. It should be noted that the possibility that the Gs-alpha pathway may be involved in the regulation of ES cell pluripotency has been suggested before. Specifically, a previous study in ES cells suggested a role for cAMP in ES cell self renewal [35]. Our finding that CTX induced the phosphorylation of CREB in ES cells suggests that Gs-alpha signaling activates the cAMP pathway in ES cells. Thus, the present study further supports the idea that the Gs-alpha-cAMP cascade may contribute to the maintenance of ES cell pluripotency.

Because GPCR signaling has received little attention in ES cells, this study reports the first direct exploration of G protein signaling and GPCR expression in ES cells. Expression profiling of GPCRs in ES cells demonstrated expression of a large number of GPCRs whose role in ES cell biology remains largely uninvestigated. Hundreds of GPCRs exist, but they signal through only 20 alpha subunits. Thus, one approach to examine GPCR signaling is to focus on signaling through one of the alpha subunits [36]. Using this rationale, we examined the impact of signaling through Gs-alpha on ES cells and report for the first time that the Gs-alpha pathway is present, functional in ES cells, increases ES cell proliferation, and impacts the expression of transcription factors important for ES cell pluripotency. This initial examination of GPCR expression and signaling in ES cells suggests a potentially important role for this family of receptors in ES cell biology. More broadly, these data indicate that this important class of signaling molecules needs to be further explored in ES cells as their activation may be important for modulating ES cells differentiation and the development of ES cell based therapies.

## Supporting Information

Figure S1 Level of mRNAs encoding proteins characteristic of ectoderm, endoderm and mesoderm. Relative changes (means ±SEM) in the level of mRNA encoding: (A) E-Cadherin (n = 3), (B) Brachyury (n = 4, blue bars) and SOX17 (n = 2, red bars), and (C) MIXL1 (n = 2, blue bars), CXCR4 (n = 3, red bars), and FOX2a (n = 3, yellow bars) in CTX-treated compared to control EBs at day 12, 16, and 20, as determined by real time RT-PCR.
(0.08 MB TIF)

Text S1 A list of the GPCR genes in the real time RT-PCR microarray.
(0.03 MB XLS)

Text S2 Raw expression data for the GPCR genes in the array.
(0.07 MB XLS)
