# Supplementary figures and images for: G Protein Coupled Receptors in Embryonic Stem Cells: A Role for Gs-Alpha Signaling
Source: PLoS One. 2010 Feb 8;5(2):e9105. doi: 10.1371/journal.pone.0009105 (PMC2816999; doi:10.1371/journal.pone.0009105)

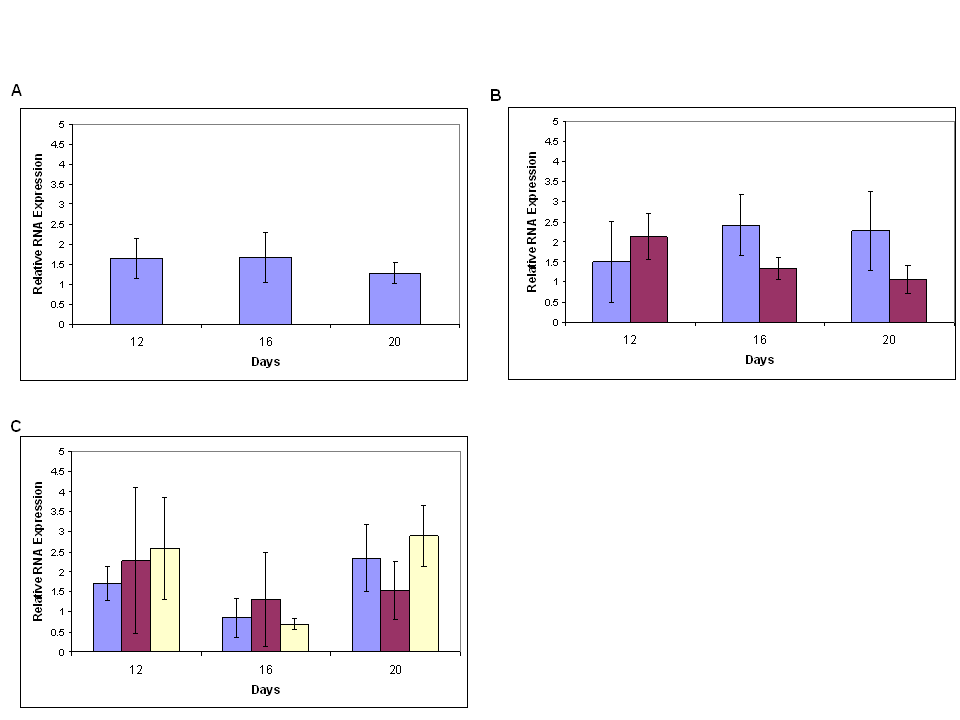

Supplement: Figure S1 — Level of mRNAs encoding proteins characteristic of ectoderm, endoderm and mesoderm. Relative changes (means ±SEM) in the level of mRNA encoding: (A) E-Cadherin (n = 3), (B) Brachyury (n = 4, blue bars) and SOX17 (n = 2, red bars), and (C) MIXL1 (n = 2, blue bars), CXCR4 (n = 3, red bars), and FOX2a (n = 3, yellow bars) in CTX-treated compared to control EBs at day 12, 16, and 20, as determined by real time RT-PCR. (0.08 MB TIF) [file pone.0009105.s001.tif]
